# *N*-(3-Methyl­phen­yl)-*N*′-(4-nitro­benzo­yl)thio­urea

**DOI:** 10.1107/S1600536808029425

**Published:** 2008-09-20

**Authors:** Liang Xian

**Affiliations:** a Chemical Engineering Institute, Northwest University for Nationalities, Lanzhou, 730030, People’s Republic of China

## Abstract

Two mol­ecules of the title compound, C_15_H_13_N_3_O_3_S, are linked by an inter­molecular N—H⋯S hydrogen bond. There is also an intra­molecular N—H⋯O hydrogen bond, forming a six-membered ring. The steric restriction of the *m*-methyl and *p*-nitro groups, as well as the intra­molecular hydrogen bond, are the main factors influencing the mol­ecular conformation.

## Related literature

For general background, see: Su *et al.* (2006 ▶). For related coordination compounds, see: Su *et al. *(2005 ▶); Xian *et al.* (2004 ▶). For related structures, see: Su (2005 ▶, 2007 ▶); Yusof *et al.* (2007 ▶).
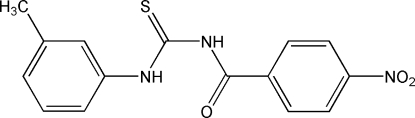


## Experimental

### 

#### Crystal data

C_15_H_13_N_3_O_3_S
*M* *_r_* = 315.34
Monoclinic, 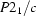

*a* = 11.381 (10) Å
*b* = 8.549 (8) Å
*c* = 15.653 (12) Å
β = 108.012 (16)°
*V* = 1448 (3) Å^3^
*Z* = 4
Mo *K*α radiation
μ = 0.24 mm^−1^
*T* = 296 (2) K
0.30 × 0.29 × 0.26 mm

#### Data collection

Bruker APEXII CCD area-detector diffractometer
Absorption correction: multi-scan (*SADABS*; Sheldrick, 2000 ▶) *T*_min_ = 0.609, *T*_max_ = 1.000 (expected range = 0.572–0.940)
7125 measured reflections
2692 independent reflections
2072 reflections with *I* > 2σ(*I*)
*R*_int_ = 0.059

#### Refinement

*R*[*F* ^2^ > 2σ(*F* ^2^)] = 0.045
*wR*(*F* ^2^) = 0.141
*S* = 0.89
2692 reflections
201 parameters
H-atom parameters constrained
Δρ_max_ = 0.29 e Å^−3^
Δρ_min_ = −0.22 e Å^−3^

### 

Data collection: *APEX2* (Bruker, 2001 ▶); cell refinement: *APEX2* and *SAINT*  (Bruker, 2001 ▶); data reduction: *SAINT*; program(s) used to solve structure: *SHELXS97* (Sheldrick, 2008 ▶); program(s) used to refine structure: *SHELXL97* (Sheldrick, 2008 ▶); molecular graphics: *SHELXTL* (Sheldrick, 2008 ▶); software used to prepare material for publication: *SHELXTL*.

## Supplementary Material

Crystal structure: contains datablocks global, I. DOI: 10.1107/S1600536808029425/bv2107sup1.cif

Structure factors: contains datablocks I. DOI: 10.1107/S1600536808029425/bv2107Isup2.hkl

Additional supplementary materials:  crystallographic information; 3D view; checkCIF report

## Figures and Tables

**Table 1:** Hydrogen-bond geometry (Å, °)

*D*—H⋯*A*	*D*—H	H⋯*A*	*D*⋯*A*	*D*—H⋯*A*
N2—H2′⋯S1^i^	0.86	2.81	3.665 (4)	179
N4—H4′⋯O3	0.86	1.94	2.643 (3)	138

Symmetry code: (i) 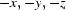.

**Fig. 1.**
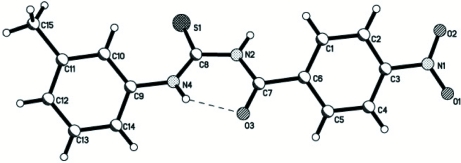
The molecular structure of the title compound. Displacement ellipsoids are drawn at the 30% probability level. The intramolecular hydrogen bonds is indicated by dashed lines.

**Fig. 2.**
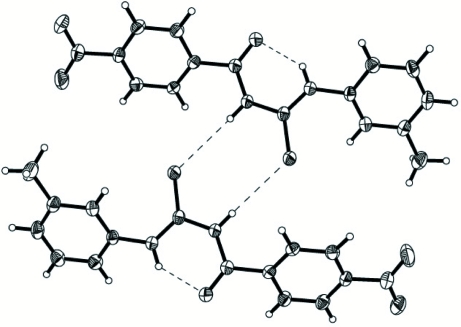
View of the dimer of the title compound formed by intermolecular hydrogen bonds (shown as dashed lines).

## supplementary crystallographic information

### Comment

Thiourea and its derivatives are good ligands for forming coordination compounds with transition metal ions, especially Cu(I). Our previous research showed that coordination compounds of carbonylthiourea derivatives with Cu(I) often adopt a trigonal planar conformation (Xian *et al.*, 2004). In addition, it was found that the reaction of carbonylthiourea derivatives with Cu(I) can also form a metal cluster compound with a complex structure (Su *et al.*, 2005). Apparently, the coordinating ability of carbonylthiorea derivatives is related to their conformation and hydrogen bonds. Herein the structure of *N*-*p*-nitrobenzoyl-*N*'-(*m*-methylphenyl)thiourea and its FT—IR, ^1^H NMR was reported.

As shown in Fig. 1, the title compound adopts a *trans*-conformation similar to the other structures of thiourea derivatives (Su *et al.*, 2006; Su, 2007), *i.e.* the conformation in which the thiocarbonyl and carbonyl groups are distributed on opposite sides of the main backbone due to steric restriction. On the other hand, steric restriction and hydrogen bond interactions also result in dimer formation through the "head-tail" junction conformation of the title compound (Fig. 2). The thiocarbonyl group forms an intermolecular hydrogen bond with N—H (-*x*, -*y*, -*z*), and the carbonyl group forms intramolecular hydrogen bond with N—H (*x*, *y*, *z*). Apparently, the carbonyl oxygen atom is "locked" in the hydrogen-bonded six-membered ring structure and thus not readily available for coordination with transition metal ions. There are mainly two molecular planes in the structure, two benzene rings almost are in the same plane with the mean deviation 0.078 (4) Å, another plane is the hydrogen-bonding six-membered ring with the mean deviation 0.055 (4) Å. The angle between two benzene planes is 41.39(0.09)°. The above conformation is similar to that observed in previously reported thiourea structures (Su, 2005; Yusof *et al.*, 2007).

### Experimental

All chemicals used for the preparation of the title compound were of reagent grade quality. The infrared spectrum was recorded in the range of 4000–400 cm^-1^ on a Nicolet NEXUS 670 F T—IR spectrometer, using KBr pellets. ^1^H NMR spectrum was obtained on an INOVA-400 MHz superconducting spectrometer, CDCl_3_ was used as the solvent and TMS as internal standard, and the chemical shifts are expressed as delta. Elemental analyses were carried out on a PE-2400 elemental analysis instrument. Melting point determination was performed in YRT-3 melting point instrument (Tianjin) and was uncorrected. The yellow single-crystal was obtained after one week by slow evaporation of the acetone solution of the title compound. *N*-*p*-nitrobenzoyl-*N*'-(*m*-methylphenyl)thiourea. Color: yellow. Melting Point: 151–153 (°C). Elemental analysis (%) found (calcd.): C, 56.3(61.5); H, 4.11(4.7); N, 10.3(13.2); S, 10.2(10.0). IR (KBr, cm^-1^): 3244 (N—H), 1675 (C=O), 1521(C=C), 1336, 1264(C=S), 1151. ^1^H NMR(delta, p.p.m.): 2.40 (s, 3H, CH_3_); 6.91–9.07 (m, 8H, C_6_H_4_, C_6_H_4_); 12.30 (s, 1H, NH).

### Refinement

The amino hydrogen atoms were found from Fourier difference maps and fixed with N—H bond lengths of 0.86 Å. The H atoms of the aromatic group were geometrically idealized. The methyl H atoms were idealized to tetrahedral geometry and allowed to freely rotate about the C-C vector. All the H atoms were refined isotropically with isotropic vibration parameters related to the atoms to which they are bonded.

### Crystal data

**Table:**

C_15_H_13_N_3_O_3_S	*F*(000) = 656
*M**_r_* = 315.34	*D*_x_ = 1.446 Mg m^−^^3^
Monoclinic, *P*2_1_/*c*	Mo *K*α radiation, λ = 0.71073 Å
*a* = 11.381 (10) Å	Cell parameters from 2974 reflections
*b* = 8.549 (8) Å	θ = 2.7–29.0°
*c* = 15.653 (12) Å	µ = 0.24 mm^−^^1^
β = 108.012 (16)°	*T* = 296 K
*V* = 1448 (3) Å^3^	Block, yellow
*Z* = 4	0.30 × 0.29 × 0.26 mm

### Data collection

**Table:**

Bruker APEXII CCD area-detector diffractometer	2692 independent reflections
Radiation source: fine-focus sealed tube	2072 reflections with *I* > 2σ(*I*)
graphite	*R*_int_ = 0.059
φ and ω scans	θ_max_ = 25.5°, θ_min_ = 1.9°
Absorption correction: multi-scan (SADABS; Sheldrick, 2000)	*h* = −13→13
*T*_min_ = 0.609, *T*_max_ = 1.000	*k* = −5→10
7125 measured reflections	*l* = −18→18

### Refinement

**Table:**

Refinement on *F*^2^	Secondary atom site location: difference Fourier map
Least-squares matrix: full	Hydrogen site location: inferred from neighbouring sites
*R*[*F*^2^ > 2σ(*F*^2^)] = 0.046	H-atom parameters constrained
*wR*(*F*^2^) = 0.141	*w* = 1/[σ^2^(*F*_o_^2^) + (0.1*P*)^2^ + 0.161*P*] where *P* = (*F*_o_^2^ + 2*F*_c_^2^)/3
*S* = 0.89	(Δ/σ)_max_ < 0.001
2692 reflections	Δρ_max_ = 0.29 e Å^−^^3^
201 parameters	Δρ_min_ = −0.22 e Å^−^^3^
0 restraints	Extinction correction: SHELXL, Fc^*^=kFc[1+0.001xFc^2^λ^3^/sin(2θ)]^-1/4^
Primary atom site location: structure-invariant direct methods	Extinction coefficient: 0.038 (4)

### Special details

**Table:**

Geometry. All e.s.d.'s (except the e.s.d. in the dihedral angle between two l.s. planes) are estimated using the full covariance matrix. The cell e.s.d.'s are taken into account individually in the estimation of e.s.d.'s in distances, angles and torsion angles; correlations between e.s.d.'s in cell parameters are only used when they are defined by crystal symmetry. An approximate (isotropic) treatment of cell e.s.d.'s is used for estimating e.s.d.'s involving l.s. planes.
Refinement. Refinement of *F*^2^ against ALL reflections. The weighted *R*-factor *wR* and goodness of fit *S* are based on *F*^2^, conventional *R*-factors *R* are based on *F*, with *F* set to zero for negative *F*^2^. The threshold expression of *F*^2^ > σ(*F*^2^) is used only for calculating *R*-factors(gt) *etc*. and is not relevant to the choice of reflections for refinement. *R*-factors based on *F*^2^ are statistically about twice as large as those based on *F*, and *R*- factors based on ALL data will be even larger.

### Fractional atomic coordinates and isotropic or equivalent isotropic displacement parameters (Å^2^)

**Table:**

	*x*	*y*	*z*	*U*_iso_*/*U*_eq_	
S1	0.16111 (5)	0.07187 (6)	0.10213 (3)	0.0457 (2)	
C6	−0.05997 (17)	0.3452 (2)	−0.17937 (12)	0.0385 (5)	
C8	0.14304 (16)	0.2443 (2)	0.05209 (11)	0.0368 (5)	
N2	0.05371 (14)	0.25694 (19)	−0.03121 (10)	0.0395 (4)	
H2'	0.0037	0.1792	−0.0469	0.047*	
C9	0.30022 (17)	0.3945 (2)	0.16605 (12)	0.0379 (5)	
C7	0.03499 (18)	0.3776 (3)	−0.09162 (13)	0.0413 (5)	
O3	0.09358 (15)	0.4980 (2)	−0.07658 (10)	0.0612 (5)	
N4	0.20710 (15)	0.3727 (2)	0.08184 (10)	0.0416 (4)	
H4'	0.1908	0.4522	0.0464	0.050*	
C10	0.28304 (18)	0.3445 (3)	0.24483 (12)	0.0427 (5)	
H10	0.2111	0.2913	0.2433	0.051*	
N1	−0.30000 (18)	0.2241 (2)	−0.43126 (12)	0.0545 (5)	
C3	−0.21803 (18)	0.2731 (2)	−0.34317 (12)	0.0406 (5)	
C1	−0.16868 (17)	0.2707 (2)	−0.18569 (12)	0.0391 (5)	
H1	−0.1879	0.2449	−0.1338	0.047*	
C2	−0.24957 (18)	0.2339 (2)	−0.26833 (13)	0.0422 (5)	
H2	−0.3239	0.1835	−0.2733	0.051*	
C12	0.4762 (2)	0.4543 (3)	0.32628 (15)	0.0544 (6)	
H12	0.5374	0.4737	0.3804	0.065*	
C11	0.37229 (19)	0.3729 (3)	0.32641 (13)	0.0465 (5)	
C5	−0.03345 (18)	0.3910 (3)	−0.25606 (13)	0.0465 (5)	
H5	0.0384	0.4468	−0.2515	0.056*	
C4	−0.11358 (19)	0.3537 (3)	−0.33902 (13)	0.0470 (5)	
H4	−0.0967	0.3830	−0.3912	0.056*	
C14	0.40313 (18)	0.4767 (3)	0.16660 (14)	0.0479 (5)	
H14	0.4140	0.5118	0.1133	0.058*	
C13	0.49105 (19)	0.5068 (3)	0.24843 (16)	0.0539 (6)	
H13	0.5613	0.5639	0.2501	0.065*	
O1	−0.27911 (18)	0.2717 (3)	−0.49730 (10)	0.0787 (6)	
O2	−0.38159 (19)	0.1337 (3)	−0.43392 (12)	0.0918 (7)	
C15	0.3541 (3)	0.3154 (4)	0.41178 (14)	0.0725 (8)	
H15A	0.3351	0.2057	0.4065	0.109*	
H15B	0.2872	0.3715	0.4228	0.109*	
H15C	0.4284	0.3321	0.4607	0.109*	

### Atomic displacement parameters (Å^2^)

**Table:**

	*U*^11^	*U*^22^	*U*^33^	*U*^12^	*U*^13^	*U*^23^
S1	0.0441 (4)	0.0420 (4)	0.0431 (3)	0.0008 (2)	0.0017 (2)	0.0030 (2)
C6	0.0384 (10)	0.0398 (11)	0.0356 (10)	0.0041 (9)	0.0088 (8)	0.0034 (9)
C8	0.0325 (9)	0.0458 (12)	0.0321 (9)	0.0017 (8)	0.0100 (8)	−0.0033 (8)
N2	0.0390 (9)	0.0397 (9)	0.0346 (8)	−0.0011 (7)	0.0039 (7)	0.0009 (7)
C9	0.0325 (9)	0.0403 (11)	0.0369 (10)	0.0032 (8)	0.0050 (8)	−0.0037 (9)
C7	0.0398 (11)	0.0424 (11)	0.0392 (10)	−0.0006 (9)	0.0083 (8)	0.0004 (9)
O3	0.0644 (10)	0.0559 (10)	0.0488 (9)	−0.0186 (9)	−0.0035 (7)	0.0098 (8)
N4	0.0423 (9)	0.0412 (10)	0.0357 (8)	−0.0034 (8)	0.0038 (7)	0.0016 (8)
C10	0.0379 (10)	0.0470 (12)	0.0408 (11)	−0.0010 (9)	0.0086 (8)	−0.0046 (10)
N1	0.0497 (11)	0.0658 (13)	0.0411 (10)	0.0018 (10)	0.0036 (8)	−0.0009 (9)
C3	0.0378 (10)	0.0457 (12)	0.0339 (10)	0.0052 (9)	0.0045 (8)	−0.0005 (9)
C1	0.0433 (11)	0.0407 (11)	0.0342 (9)	0.0019 (9)	0.0134 (8)	0.0035 (9)
C2	0.0349 (10)	0.0448 (11)	0.0458 (11)	0.0011 (9)	0.0108 (8)	0.0029 (9)
C12	0.0398 (12)	0.0633 (15)	0.0474 (12)	0.0050 (11)	−0.0051 (9)	−0.0084 (11)
C11	0.0470 (12)	0.0497 (12)	0.0379 (10)	0.0091 (10)	0.0058 (9)	−0.0015 (10)
C5	0.0362 (10)	0.0610 (14)	0.0415 (11)	−0.0053 (10)	0.0110 (9)	0.0039 (10)
C4	0.0450 (11)	0.0609 (14)	0.0365 (10)	0.0010 (10)	0.0145 (9)	0.0052 (10)
C14	0.0391 (11)	0.0546 (13)	0.0490 (12)	0.0009 (10)	0.0120 (9)	0.0014 (10)
C13	0.0313 (10)	0.0620 (15)	0.0617 (13)	−0.0068 (10)	0.0046 (9)	−0.0052 (12)
O1	0.0929 (14)	0.0997 (16)	0.0343 (8)	−0.0134 (12)	0.0062 (8)	0.0072 (9)
O2	0.0754 (12)	0.1309 (19)	0.0583 (11)	−0.0485 (14)	0.0046 (9)	−0.0138 (12)
C15	0.0825 (18)	0.090 (2)	0.0401 (12)	−0.0026 (16)	0.0122 (12)	0.0013 (13)

### Geometric parameters (Å, °)

**Table:**

S1—C8	1.652 (3)	C3—C4	1.358 (3)
C6—C1	1.368 (3)	C3—C2	1.369 (3)
C6—C5	1.382 (3)	C1—C2	1.372 (3)
C6—C7	1.488 (3)	C1—H1	0.9300
C8—N4	1.320 (3)	C2—H2	0.9300
C8—N2	1.388 (2)	C12—C13	1.358 (4)
N2—C7	1.371 (3)	C12—C11	1.372 (3)
N2—H2'	0.8600	C12—H12	0.9300
C9—C14	1.364 (3)	C11—C15	1.497 (3)
C9—C10	1.375 (3)	C5—C4	1.374 (3)
C9—N4	1.425 (3)	C5—H5	0.9300
C7—O3	1.210 (3)	C4—H4	0.9300
N4—H4'	0.8600	C14—C13	1.384 (3)
C10—C11	1.385 (3)	C14—H14	0.9300
C10—H10	0.9300	C13—H13	0.9300
N1—O2	1.199 (3)	C15—H15A	0.9600
N1—O1	1.201 (3)	C15—H15B	0.9600
N1—C3	1.467 (3)	C15—H15C	0.9600
			
C1—C6—C5	120.32 (18)	C2—C1—H1	119.9
C1—C6—C7	122.42 (18)	C3—C2—C1	118.3 (2)
C5—C6—C7	117.25 (19)	C3—C2—H2	120.9
N4—C8—N2	115.52 (18)	C1—C2—H2	120.9
N4—C8—S1	126.90 (15)	C13—C12—C11	121.0 (2)
N2—C8—S1	117.56 (15)	C13—C12—H12	119.5
C7—N2—C8	128.27 (18)	C11—C12—H12	119.5
C7—N2—H2'	115.9	C12—C11—C10	118.3 (2)
C8—N2—H2'	115.9	C12—C11—C15	121.7 (2)
C14—C9—C10	120.97 (18)	C10—C11—C15	120.1 (2)
C14—C9—N4	117.68 (18)	C4—C5—C6	119.7 (2)
C10—C9—N4	121.17 (19)	C4—C5—H5	120.1
O3—C7—N2	123.19 (19)	C6—C5—H5	120.1
O3—C7—C6	122.51 (19)	C3—C4—C5	118.59 (19)
N2—C7—C6	114.24 (18)	C3—C4—H4	120.7
C8—N4—C9	127.32 (17)	C5—C4—H4	120.7
C8—N4—H4'	116.3	C9—C14—C13	118.4 (2)
C9—N4—H4'	116.3	C9—C14—H14	120.8
C9—C10—C11	120.3 (2)	C13—C14—H14	120.8
C9—C10—H10	119.8	C12—C13—C14	120.9 (2)
C11—C10—H10	119.8	C12—C13—H13	119.5
O2—N1—O1	123.1 (2)	C14—C13—H13	119.5
O2—N1—C3	118.5 (2)	C11—C15—H15A	109.5
O1—N1—C3	118.3 (2)	C11—C15—H15B	109.5
C4—C3—C2	122.73 (18)	H15A—C15—H15B	109.5
C4—C3—N1	118.87 (18)	C11—C15—H15C	109.5
C2—C3—N1	118.4 (2)	H15A—C15—H15C	109.5
C6—C1—C2	120.21 (18)	H15B—C15—H15C	109.5
C6—C1—H1	119.9		
			
N4—C8—N2—C7	9.9 (3)	C5—C6—C1—C2	3.3 (3)
S1—C8—N2—C7	−168.46 (16)	C7—C6—C1—C2	−175.48 (19)
C8—N2—C7—O3	−4.2 (3)	C4—C3—C2—C1	−3.5 (3)
C8—N2—C7—C6	173.12 (17)	N1—C3—C2—C1	175.73 (18)
C1—C6—C7—O3	−141.1 (2)	C6—C1—C2—C3	0.2 (3)
C5—C6—C7—O3	40.1 (3)	C13—C12—C11—C10	0.5 (3)
C1—C6—C7—N2	41.5 (3)	C13—C12—C11—C15	−179.4 (2)
C5—C6—C7—N2	−137.3 (2)	C9—C10—C11—C12	1.2 (3)
N2—C8—N4—C9	177.28 (17)	C9—C10—C11—C15	−178.8 (2)
S1—C8—N4—C9	−4.5 (3)	C1—C6—C5—C4	−3.6 (3)
C14—C9—N4—C8	138.7 (2)	C7—C6—C5—C4	175.16 (19)
C10—C9—N4—C8	−46.1 (3)	C2—C3—C4—C5	3.1 (3)
C14—C9—C10—C11	−2.0 (3)	N1—C3—C4—C5	−176.1 (2)
N4—C9—C10—C11	−177.11 (18)	C6—C5—C4—C3	0.5 (3)
O2—N1—C3—C4	169.0 (2)	C10—C9—C14—C13	1.0 (3)
O1—N1—C3—C4	−8.1 (3)	N4—C9—C14—C13	176.2 (2)
O2—N1—C3—C2	−10.3 (3)	C11—C12—C13—C14	−1.5 (4)
O1—N1—C3—C2	172.6 (2)	C9—C14—C13—C12	0.8 (4)

### Hydrogen-bond geometry (Å, °)

**Table:**

*D*—H···*A*	*D*—H	H···*A*	*D*···*A*	*D*—H···*A*
N2—H2'···S1^i^	0.86	2.81	3.665 (4)	179.
N4—H4'···O3	0.86	1.94	2.643 (3)	138.

Symmetry codes: (i) −*x*, −*y*, −*z*.
